# Exploring the construction of a youth mental health campus ecosystem integrating research-practice teaching

**DOI:** 10.3389/fpubh.2025.1636327

**Published:** 2025-07-23

**Authors:** Tao Gao

**Affiliations:** Mental Health Education and Counseling Center, Guangdong Industry Polytechnic University, Guangzhou, China

**Keywords:** youth mental health, research-practice teaching, ecological system, collaborative education, campus ecosystem

## Abstract

This study explores the construction of a youth mental health-promoting campus ecosystem based on integrated research-practice teaching. In response to the fragmentation of traditional mental health education in Chinese universities, the model incorporates ecosystem theory, embodied cognition, and multi-agent collaboration to build a four-dimensional support system: curriculum-practice integration, internal-external stakeholder synergy, environmental optimization, and institutional sustainability. Through immersive practices like psychodrama, mind mapping, and mindful walking, students’ psychological capital and self-regulation abilities are enhanced. The model emphasizes collaboration across psychological, educational, and managerial units, as well as coordination with families, communities, medical institutions, and enterprises. By combining physical space design with digital tracking platforms, the ecosystem enables all-scenario psychological support. Challenges such as insufficient longitudinal validation, ethical concerns in AI-driven interventions, and uneven resource allocation remain. This study provides a localized and actionable framework for advancing youth mental health in higher education, supporting a shift from crisis intervention to positive psychological development.

## 1 Introduction

In recent years, the mental health issues among the youth population have become a major public health concern worldwide. Especially since the outbreak of the COVID-19 pandemic, the psychological condition of young people has further deteriorated, with prominent symptoms such as depression, anxiety, loneliness, and social withdrawal (1). The pandemic and its resulting academic anxiety, employment pressure, and life uncertainties have made college students’ mental health issues particularly prominent. Many students exhibit obvious tendencies of anxiety and depression (2), even leading to severe psychological crises (3). In China, with the expansion of college enrollment and increased academic pressure, along with social transformation and tight employment situations, college students’ mental health problems show characteristics such as “unclear responsibilities, high pressure, and significant difficulty.” The demand for psychological crisis intervention and support services continues to rise (4).

However, the current mental health education system in colleges and universities still mainly relies on traditional counseling room services and theoretical classroom teaching. It emphasizes intervention over prevention and lacks collaboration between medical and educational institutions, resulting in significant fragmentation that fails to adequately respond to the diverse and developmental psychological needs of young students (5). On the one hand, mental health education often remains at the level of theoretical knowledge transmission, lacking practical, experiential, and contextual teaching design, leading to insufficient attention from students and low participation rates (6). On the other hand, the collaborative mechanisms for mental health education in colleges have not been effectively established. The linkage system among families, schools, communities, enterprises, and medical institutions has yet to be formed, and insufficient resource sharing and collaboration hinder the full effectiveness of education (5, 7, 8).

In view of this, ecosystem thinking has gradually been introduced into the field of mental health education in higher education. Ecosystem theory emphasizes that individual development is influenced by interactions within multi-level environments. From this theoretical perspective, mental health education advocates a comprehensive collaborative model, integrating educational resources, institutional design, and the natural environment to form a student-centered, holistic psychological support system (9, 10).

At the same time, research-practice teaching, as an innovative pedagogical approach that centers on “experiential research-practice + psychological cultivation,” has increasingly attracted the attention of educational researchers. It emphasizes students’ participation in immersive experiential practices such as psychodrama, mind maps, outdoor development, and mindfulness training, thereby promoting the accumulation of psychological capital and activation of positive emotions (11, 12). Through process-oriented, experiential learning and active student participation, this approach effectively stimulates students’ intrinsic motivation, cognitive experiences, and behavioral transformation, providing methodological support for constructing an ecosystem-based mental health education system (13, 14).

Internationally, institutions represented by the World Health Organization (WHO) have proposed the Youth-Friendly Health Services Framework, which emphasizes that mental health services should be characterized by accessibility, appropriateness, acceptability, equity, and effectiveness (15). For example, Singapore’s CHAT (Community Health Assessment Team), as well as campus ecosystem service models in parts of India and the United States, integrate school, family, community, and medical resources to form highly efficient mental health ecosystems (1, 16). Compared with these advanced international models, the construction of mental health ecosystems in Chinese universities is still at an early stage, lacking systematic practical cases and methodological guidance, urgently requiring innovative local models and empirical validation.

The youth mental health campus ecosystem that integrates research-practice teaching aims to explore an ecological mental health education path that is more feasible, effective, and sustainable. By integrating ecosystem theory, positive psychology, embodied cognition theory, and multi-stakeholder collaboration theory, this study attempts to systematically address the current fragmentation in mental health education in higher education. It seeks to build a dynamically balanced system that encompasses “curriculum–practice–support–environment,” and further proposes a four-dimensional ecological construction path involving curriculum-practice integration, multi-agent collaboration, comprehensive environmental optimization, and long-term institutional assurance.

## 2 Related research on the youth mental health campus ecosystem integrating research-practice teaching

Currently, the psychological development of college students and the construction of mental health campus ecosystems have become cutting-edge topics in the field of educational psychology worldwide. Both domestic and international research has conducted in-depth exploration on individual psychological development of college students, construction of campus psychological support systems, and innovation in teaching models from various perspectives.

The integration of research-practice teaching is a pedagogical model that organizes students into collaborative learning groups and adopts teaching methods that stimulate student interest. In this model, teachers act as organizers who proactively establish systematic connections with families, society, enterprises, and hospitals to better serve teaching through the integration of multi-agent collaborative resources. It is a teaching approach aimed at developing student competencies and enhancing teaching effectiveness. Through various immersive learning forms such as psychodrama, mind mapping, and outdoor development, students achieve psychological growth by “learning through doing.” This approach has been validated by multiple empirical studies for its effective implementation (11, 12).

The campus psychological ecological environment refers to the introduction of the concept of ecological environment into school education, specifically the creation of a campus environment conducive to students’ mental health development (13, 14). A mental health campus ecosystem is defined as a student-centered multidimensional support system that dynamically balances teaching, environment, institutional mechanisms, and social resources. It emphasizes comprehensiveness, systematicity, and sustainability (13).

### 2.1 International research

McDaid and colleagues have pointed out that investments in adolescent mental health interventions yield substantial returns, with health and economic benefits reaching up to 24 times the cost (17). For young adults, interventions that teach coping skills for common psychological problems—such as procrastination, perfectionism, low self-esteem, test anxiety, and stress—may reduce the incidence of specific mental health disorders, while also offering more accessible and non-stigmatizing care options for youth (18, 19).

In recent years, research on urban and campus ecosystems has increasingly converged, focusing on how environmental factors contribute to youth mental health. Collins et al. (20) suggested that constructing green spaces and interactive natural environments can significantly enhance the mental well-being of urban residents and students. Enhancing youth mental health requires a holistic ecological perspective that integrates individual growth environments, social support systems, and educational resources. Studies from Western countries, notably in Europe and North America, have emphasized the “developmental” and “ecosystemic” nature of psychological education, arguing that multi-level collaboration and support—from individuals and families to society—can effectively prevent psychological issues in youth, strengthen emotional regulation, self-efficacy, and social connectedness, and significantly reduce the prevalence of internalizing behavioral problems such as depression and anxiety (20). Buxton et al. (9) further confirmed that natural soundscapes in urban environments have positive effects in alleviating anxiety and improving mental health, underscoring that campus environment design should integrate ecological landscapes and experiential practices to create physical spaces conducive to mental well-being.

### 2.2 Current research in China

In recent years, domestic research has made significant progress in the following three areas:

#### 2.2.1 Blended curriculum design

Blended curriculum design innovatively combines online autonomous learning with in-person classroom instruction. During online sessions, students utilize digital learning platforms to preview course content independently and construct a knowledge framework in advance. Offline classes then focus on deep interaction, using activities such as case analysis and group discussion to closely integrate theory with practice and enhance students’ comprehension and mastery of course material. At the same time, based on students’ personalized learning needs and characteristics, the courses flexibly employ various teaching methods such as case-based learning, group collaboration, and role-playing to fully stimulate student engagement, effectively boost learning motivation, and improve learning outcomes and teaching quality.

#### 2.2.2 Emergence of research hotspots in campus mental health ecosystem construction

Researchers such as Liu Fushu and Wu Xietao have proposed that mental health education should be implemented through campus environmental planning, cultural construction, and institutional optimization (13, 21). Wu Tong further pointed out that the key to building a campus mental health ecosystem lies in the cultivation of psychological education professionals and the establishment of a multi-agent collaborative operational mechanism (22). However, existing domestic studies still lack in-depth exploration of the specific operational mechanisms of multi-agent collaboration, and practical implementation pathways remain underdeveloped.

#### 2.2.3 Innovation and transformation in mental health education models

Some domestic scholars have actively explored new models for mental health education. For instance, Gao and colleagues proposed the “research-practice teaching model,” which incorporates psychodrama, mind mapping, and blended learning methods to effectively enhance student interest and psychological adjustment capabilities (23–25). Meanwhile, the evaluation methods for mental health education have also undergone transformation and innovation. The “dual-subject intersex collaborative evaluation” mechanism suggests conducting teaching evaluations through teacher-student mutual evaluation to better improve teaching (26).

#### 2.2.4 Integration of social support systems

Social support systems play a crucial role in psychological support. The family, as an essential component of the social support system, can better leverage its strengths through effective home-school collaboration mechanisms. This allows for mutual complementation of educational strategies and ensures the scientific and effective implementation of mental health education. It also facilitates the temporal and spatial extension of psychological education and optimizes educational outcomes (8). At the same time, the effective integration of resources from enterprises, medical institutions, and other societal sectors provides broader support spaces and resource guarantees for mental health education in universities. For example, Wang (27) noted that school-enterprise collaboration can effectively foster a healthy and positive social mentality, thereby enabling mutual promotion between students’ psychological and career development. Domestic research has also made important advances in medical-school collaboration. For instance, Wuhan University proposed the “Preventive Medicine” model of collaboration between medical and educational institutions, emphasizing the integration of psychological health prevention and intervention strategies (28).

Research-practice teaching promotes the integration of social resources by inviting parents, enterprises, medical professionals, and community partners to participate in collaborative research-practice activities. This fosters educational synergy, extends the temporal and spatial continuity of the youth mental health campus ecosystem, and significantly enhances teaching effectiveness.

Nevertheless, current studies on the “research-practice model” in mental health education still lack comprehensive and systemic application. Existing research mostly involves only partial stakeholder collaboration. The integration of research-practice teaching still requires deeper and more extensive exploration.

## 3 The ecological dilemma of traditional campus mental health education

Although in recent years, the mental health education system in Chinese universities has gradually improved, significant structural deficiencies remain in its practical implementation. These issues constrain the effectiveness and sustainability of campus mental health education. The ecological dilemma of traditional campus mental health education is mainly reflected in the following two aspects.

### 3.1 Tructural imbalance

#### 3.1.1 Disconnection between curriculum and practice

Currently, mental health education in universities is primarily theoretical. Teachers tend to focus on explaining basic psychological concepts and disseminating mental health knowledge, while neglecting the connection between students’ actual psychological needs and real-life scenarios. The course content is often abstract and homogeneous, with a severe lack of practical, research-practice-oriented courses, resulting in students being unable to effectively transform the acquired theoretical knowledge into practical skills for psychological adjustment (5).

Lin (13) pointed out that if a course lacks experiential learning modules and is limited to traditional lecturing by teachers, it may lead to low student engagement and unsustainable learning outcomes. Such fragmented teaching methods fail to cultivate students’ genuine psychological adjustment abilities and psychological capital.

#### 3.1.2 Lack of multi-agent collaboration

University mental health education lacks an effective collaborative system that integrates families, schools, communities, medical institutions, and enterprises. The cooperation among various stakeholders often remains superficial, without forming a genuine framework for resource sharing and in-depth interaction.

Educational resources from families and schools have not been effectively integrated. Most parents have limited psychological education capabilities and a weak sense of participation. Interactions between families and schools are usually limited to surface-level communication, and deep cooperation mechanisms and shared objectives have yet to be established (8). In addition, enterprise and medical resources have not been effectively integrated into campus mental health education systems. Consequently, universities lack professional resource support in psychological crisis intervention and career-related psychological adaptation education, leading to a severe lack of synergistic effects (7, 29).

#### 3.1.3 Inadequate environmental support

The design of physical spaces in traditional campuses often fails to align with students’ psychological needs. In most universities, psychological support spaces are limited to a few fixed areas such as counseling centers, with little attention paid to designing psychologically friendly public spaces. The lack of green areas fails to meet students’ diverse psychological needs and increases the risk of negative mental health outcomes (20).

Moreover, the application of digital psychological support technologies in universities is significantly lagging. There is a lack of infrastructure for data collection and analysis. Current psychological interventions have not yet systematically integrated digital infrastructure with psychological support services. This calls for the participation of interdisciplinary teams to build a “whole-person care” mental health ecosystem (15).

### 3.2 Adaptation challenges in mental health education

#### 3.2.1 Widespread passive participation among students

Research-practice teaching emphasizes students’ active participation and experiential engagement. However, in practical implementation, students are often accustomed to traditional passive learning modes and exhibit significant maladaptation to active experiential participation. In practice, students commonly show strong “bystander effects,” lacking enthusiasm for self-expression and deep thinking. As a result, it becomes difficult to activate the classroom atmosphere of research-practice teaching, severely compromising teaching effectiveness.

#### 3.2.2 Monolithic evaluation systems

At present, universities still predominantly use outcome-oriented traditional evaluation systems in mental health education. These systems focus on students’ memorization and comprehension of knowledge while neglecting the systematic tracking and evaluation of their psychological development processes.

Research-practice teaching is characterized by a process-oriented approach. However, current evaluation systems fail to effectively reflect students’ psychological changes, experiential feedback, and competency development during the research-practice process. This makes it difficult to objectively quantify and systematically optimize the effectiveness of such courses (26).

## 4 Constructing a youth mental health ecosystem through integrated research-practice teaching

To effectively overcome the ecological dilemmas inherent in traditional campus mental health education, constructing an ecological pathway that integrates research-practice teaching offers a promising direction. This model aims to realize a systematic transformation of mental health education in universities through the integration of curriculum and practice, multi-agent collaboration, environmental optimization, and institutional assurance.

### 4.1 Integration of curriculum and practice: building a new paradigm of research-practice teaching and its three-dimensional curriculum system

The foundational theoretical curriculum should include basic knowledge of mental health, psychological assessment techniques, and crisis intervention strategies. Courses such as College Student Mental Health Education can serve as required modules. These courses focus on constructing theoretical frameworks and enhancing students’ sensitivity to psychological problems as well as their preliminary identification skills (13, 14).

Research-practice-oriented courses emphasize experiential learning through contextualized activities such as psychodrama, group sandplay, and mindful walking. These enable students to acquire psychological adjustment skills through hands-on experiences. Gao (11) found that group psychodrama effectively helps students express internal emotions and conflicts, enhancing both empathy and psychological resilience. Technological empowerment modules—such as using mind maps in collaborative research-practice psychological adjustment training—further increase the immersive and authentic nature of learning. With the support of technology, students quickly develop embodied cognition and experience positive psychological states (11, 30).

The social practice module emphasizes collaboration between universities and enterprises to conduct psychological quality development and community-based psychological services. These help students apply psychological competencies in real-world contexts and deepen the integration between theoretical knowledge and practical scenarios (27).

### 4.2 Multi-agent collaboration: enhancing the support network of the ecosystem

#### 4.2.1 Internal collaboration: establishing a “psychology–teaching–management” community

The key to internal stakeholder collaboration lies in the development of the teaching team. Psychological counselors are responsible for the design and guidance of research-practice courses, while subject-matter teachers embed mental health content into disciplinary teaching to form a linkage mechanism. In addition, universities can organize regular training around “research-practice teaching” to enhance teachers’ experiential instructional abilities.

At the student level, a peer mentoring system can be established, forming a three-tiered support network composed of “psychological monitors–dormitory leaders–volunteers.” Within research-practice classrooms, this peer support system actively participates in teaching activities, enhancing students’ capacity for self-management and their active engagement in mental health education (13).

#### 4.2.2 External collaboration: expanding family–school–community–medical–enterprise linkage

Family-school collaboration can be promoted through initiatives such as “parent classrooms,” online psychological lectures, and offline parent–child workshops, which strengthen the integration of family education resources with school-based mental health education (7, 8). In addition, a “Family Research-Practice Log” can be established to record students’ research-practice activities at home, reinforcing the support of family social capital for students’ psychological growth.

The integration of social resources includes cooperation with medical institutions to establish “green channels” for psychological crisis referrals and the construction of a “Hospital–University–Association” tripartite linkage mechanism (29). Furthermore, the introduction of Employee Assistance Programs (EAPs) can provide career-oriented psychological adjustment training, enhancing students’ psychological adaptability both before and after entering the workforce (31).

### 4.3 Environmental reconfiguration: creating an all-scenario mental health support space

#### 4.3.1 Optimization of physical space

Universities should build dedicated “research-practice spaces,” such as mental wellness research-practice rooms, group counseling rooms, and five-sense (visual, auditory, tactile, gustatory, olfactory) sensory gardens. These specialized areas enhance the alignment between physical environments and students’ psychological adjustment needs. Increasing campus green spaces, comfortable seating areas, and artistic installations can further improve the psychological friendliness and comfort of the environment (20).

#### 4.3.2 Empowerment through digital environments

Universities should develop a “Campus Psychological Research-Practice Platform” that integrates online course learning, psychological assessment tools, and counseling appointment systems. This creates an effective connection between offline research-practice activities and online data tracking. By leveraging big data analytics to identify student behavior patterns, potential psychological risks can be predicted in a timely manner. For example, using digital health technologies (such as Wi-Fi data, browsing history, and app interventions) to monitor students’ social activity and campus life rhythms—without infringing on privacy—can facilitate proactive intervention (15).

## 5 Discussion

The youth mental health campus ecosystem constructed through the integration of research-practice teaching represents a systematic reform of the traditionally fragmented model of mental health education. By integrating ecosystem theory, embodied cognition theory, and collaboration theory, this model addresses the structural dilemmas of disconnection between curriculum and practice, insufficient stakeholder collaboration, and the singularity of environmental support in traditional education. It provides a four-dimensional solution—“Curriculum–Practice–Support–Environment”—that enables a holistic transformation of mental health education in higher education institutions.

### 5.1 Effectiveness and innovative value of research-practice teaching

Research-practice teaching, through immersive practices such as psychodrama, mind mapping, and VR technology, effectively activates students’ subjectivity and embodied cognition. For instance, the process of selecting topics and performing in psychodrama (12) not only enhances students’ emotional expression abilities but also promotes self-awareness and social empathy through role-playing. This closely aligns with the theoretical logic of positive psychology that emphasizes “enhancing psychological capital through experiential learning” (13).

Furthermore, the technology-empowered module—such as mind map-based collaborative research-practice—improves knowledge construction efficiency through visual thinking tools. Data show that over 98% of students expressed interest in this model, and 97.26% considered it effective for most or specific teaching content (32), confirming the validity of embodied cognition theory in psychological education.

### 5.2 Ecological effects of multi-stakeholder collaboration

The establishment of an internal “Psychology–Teaching–Management” community and an external “Family–School–Community–Medical–Enterprise” linkage mechanism addresses the limitation of single-agent resource dependence in traditional education. For example, the “Four-Level Psychological Protection Network” at Beijing Normal University (33) operates through hierarchical collaboration involving student leaders, departments, the university’s psychological center, and institutional governance, significantly improving early identification and intervention in psychological crises.

Externally, the “Government–Media–Experts–Parents” model of the “Yangtze River Mental Health Base” in Wuhan (34), promoted through community operation and resource integration, emphasizes full-process parental participation and highlights the enhancement of psychological support networks through multi-agent collaboration.

However, current home-school educational resources have yet to be effectively integrated (8). The in-depth involvement of enterprise and medical resources remains insufficient. Universities still face a serious lack of collaborative support in psychological crisis intervention and career-related psychological adaptation (7, 29). In the future, policy guidance is needed to clarify responsibilities and rights—for instance, incorporating enterprise-based psychological support into the assessment indicators of school-enterprise cooperation.

### 5.3 Dual dimensions of environmental reconfiguration

The optimization of physical space and the empowerment of digital technology together form an “offline immersion–online tracking” all-scenario support system. The addition of campus green spaces and natural therapeutic zones echoes international findings on the “positive correlation between biodiversity and mental health” (9). For example, the high utilization rate (98%) of open spaces at Kwame Nkrumah University of Science and Technology (KNUST) and their facilitative role in relaxation and social interaction (35) confirm the direct impact of physical environments on psychological states.

Meanwhile, the development of digital platforms breaks through temporal and spatial limitations. The “Campus Psychological Research-Practice Platform,” for instance, utilizes big data to detect risk behaviors such as social withdrawal and leverages AI psychological tutors to deliver real-time interventions. This resonates with the concept of “constructing a whole-person care ecosystem through digital health technologies” proposed by Perimal-Lewis et al. (15). However, privacy protection issues must not be overlooked. Practices from Singapore’s CHAT program—such as anonymization technology and ethical standards (1)—can be referenced to ensure compliance in technological applications.

### 5.4 Long-term challenges of institutional assurance

Innovating the evaluation system is key to the sustainable operation of the ecosystem. The “Dual-Subject Interactivity Collaborative Evaluation” model (23, 26), which integrates student self-assessment, peer evaluation, and teacher feedback, compensates for the traditional assessment system’s neglect of psychological development processes. However, practical implementation still faces challenges such as vague quantitative criteria and increased evaluation burden on instructors.

In the future, blockchain technology could be introduced to record data from the research-practice process. By combining qualitative and quantitative indicators, a dynamic assessment model can be established. Additionally, cross-departmental policy collaboration is still lacking, resulting in uneven resource distribution. Research-practice facilities in universities located in economically underdeveloped regions remain insufficient. Special funding and regional resource-sharing mechanisms (such as the “Psychological Education Consortium”) should be used to bridge these gaps.

### 5.5 Research limitations and future directions

While this study presents a systematic framework for constructing a youth mental health campus ecosystem, it currently lacks robust empirical validation. It lacks longitudinal data to verify the sustained impact of the constructed ecosystem. The findings are primarily derived from case analyses and short-term intervention outcomes, without the support of longitudinal or controlled experimental data. As such, the outcomes remain exploratory rather than conclusive. Future research should prioritize the design and implementation of large-scale, cross-institutional longitudinal studies, with an emphasis on quantifiable psychological and behavioral outcomes across diverse student populations and academic stages. The inclusion of control groups and standardized metrics of effectiveness will be essential to evaluate the replicability and impact of the model.

Implementation strategies also require further development, particularly for low-resource or underserved institutions where infrastructure and personnel for research-practice integration may be limited. Future work should offer scalable, cost-effective pathways tailored to such settings—for example, leveraging existing student services or peer support networks for program delivery. Additionally, teacher training protocols should be systematized, ensuring facilitators are equipped with both psychological and technological competencies to sustain the ecosystem’s operation.

Culturally specific interventions—such as those integrating traditional culture or calligraphy-based therapy—remain in the early stages of implementation. These approaches should be further developed using frameworks from cultural psychology theories and indigenous mental health models, with rigorous evaluation to determine their relevance and impact among targeted populations. Deep cultural adaptation will be necessary to avoid superficial integration and ensure contextual sensitivity.

From an ethics standpoint, the use of digital tools—including AI-driven platforms, behavioral tracking, and online sentiment analysis—raises critical concerns regarding data privacy, algorithmic bias, and informed consent. While these concerns are acknowledged, future work must strengthen the ethical infrastructure underpinning such technologies. This includes developing transparent algorithmic auditing processes, drawing on international standards such as the European Union’s General Data Protection Regulation (GDPR) and China’s Personal Information Protection Law. Clear protocols for data minimization, purpose limitation, and informed consent must be implemented to safeguard student rights and promote trust in psychological technologies.

Finally, to align the proposed ecosystem with international best practices, future studies should consider embedding principles from frameworks such as the WHO’s Youth-Friendly Health Services Framework (15) into China’s higher education governance structures. Concepts like accessibility and equity, and student-centeredness must be operationalized through policy innovation, aligned with national strategies such as the “Healthy China 2030” Plan. By combining global standards with localized adaptations, the model can evolve into a replicable and context-sensitive framework for advancing youth mental health in higher education.

## 6 Conclusion

The youth mental health campus ecosystem that integrates research-practice teaching provides an innovative solution to the global youth mental health crisis. Its core value lies in breaking through the fragmented limitations of traditional education by constructing a comprehensive support system along four key pathways: “activating cognition through practice, integrating resources through collaboration, cultivating growth through immersive environments, and sustaining impact through institutional assurance.” These efforts collectively build an “all-staff, all-process, all-dimensional” support system.

The practical outcomes of this model not only offer an operable paradigm for mental health education in higher education institutions, but also provide theoretical references for constructing a broader social psychological service system under the “Healthy China” national strategy.

Looking ahead, it is necessary to further promote the triadic development of “policy legislation, technological innovation, and cultural adaptation.” This includes clarifying the roles and responsibilities of all stakeholders through cross-sector policy coordination; improving the precision and effectiveness of psychological interventions through the application of AI and big data technologies; and developing localized mental health education projects rooted in traditional culture.

Only by advancing along these directions can we truly build a resilient youth mental health ecosystem. Such a system would support a paradigm shift from “problem-based intervention” to “positive development,” and construct a strong psychological safeguard for the healthy development of young people.

## Data Availability

The original contributions presented in the study are included in the article/supplementary material, further inquiries can be directed to the corresponding author.
