# Correction: Exploring the construction of a youth mental health campus ecosystem integrating research-practice teaching

**DOI:** 10.3389/fpubh.2025.1739789

**Published:** 2025-11-19

**Authors:** Tao Gao

**Affiliations:** Mental Health Education and Counseling Center, Guangdong Industry Polytechnic University, Guangzhou, China

**Keywords:** youth mental health, research-practice teaching, ecological system, collaborative education, campus ecosystem

In the published article, there was a mistake in the **Funding** statement. The funder Guangdong Provincial Department of Education 2025 Annual Higher Education Institution Research Platform and Project: Research on Teaching Practice Empowering College Students' Mental Health: Innovation of Multi-Ecological Collaborative Mechanism, Grant Number 2025WTSCX172 to Tao Gao was erroneously omitted. The corrected Funding statement appears below:

“The author(s) declare that financial support was received for the research and/or publication of this article. This work was supported by Education and Teaching Reform Project of Guangdong Industry Polytechnic University in 2023 (JG202301); Education and Teaching Achievement Award Cultivation Project of Guangdong Industry Polytechnic University in 2023 “Five in One, Six in One Education Integration: Innovative Practice of Psychological Health Collaborative Research and Training”; this paper is the research achievement of the 2025 Foshan Youth Development-Oriented City Construction Research Project (Project Code: 2025-QNFZ81). Ministry of Education Supply and Demand Matching Employment and Education Project (Phase IV) (Project Code: 2025071284192, 2025071228168). This work was supported by Guangdong Provincial Department of Education 2025 Annual Higher Education Institution Research Platform and Project: Research on Teaching Practice Empowering College Students' Mental Health: Innovation of Multi-Ecological Collaborative Mechanism (Project Code: 2025WTSCX172).”

The original version of this article has been updated.

